# Effects of Systemic Tadalafil on Skin Flap Survival in Rats

**Published:** 2012-09-12

**Authors:** Michael B. Brewer, Amy L. Stump, Luther H. Holton, Lindsay E. Janes, Ronald P. Silverman, Devinder P. Singh

**Affiliations:** Division of Plastic Surgery, Department of Surgery, University of Maryland Medical Center, Baltimore, Md

## Abstract

**Objective:** Phosphodiesterase-5 inhibitors, used to increase penile blood flow in erectile dysfunction patients, have recently been postulated to increase blood flow and flap survival in cutaneous flaps based on random blood supply. This study aims to investigate the phosphodiesterase-5 inhibitor tadalafil, administered orally, on random flap survival. **Methods:** Modified McFarlane flaps measuring 8 cm × 2.5 cm were raised on the backs of 37 male Sprague-Dawley rats. Rats received were divided into a control group, a low-dose group (10 mg/kg tadalafil), and a high-dose group (20 mg/kg tadalafil). Treatment doses were administered once preoperatively and every 24 hours postoperatively for a total of 7 doses. On postoperative day 7 and 14, the area of flap survival was calculated and compared. **Results:** All rats survived and thrived throughout the experimental period. Control group rats showed an average flap survival of 77% ± 11% at 7 days and 77% ± 9% at 14 days. Low-dose-group rats showed an average flap survival of 82% ± 10% at 7 days (*P*=0.21), and 81% ± 12% at 14 days (*P*=0.41). High-dose group rats showed an average flap survival of 81% ± 11% at 7 days (*P* = 0.45) and 80% ± 12% at 14 days (*P* = 0.53). Statistical analysis was performed using the Mann-Whitney test. **Conclusions:** Our results indicate a trend toward increased random-pattern flap survival with both high- and low-dose oral tadalafil in a rat model. Because this trend did not achieve statistical significance, further studies are warranted.

Oncologic resections and traumatic injury often cause loss of skin and subcutaneous tissues, resulting in functional and cosmetic defects. The resultant loss of the protective barrier of the skin puts the patient at risk for infection and further tissue loss. The mainstay in surgical reconstruction of such defects involves the use of local, rotational, or free flaps. In regional flap techniques, a segment of skin, subcutaneous tissue, and sometimes underlying muscle or fascia is dissected free of adjacent tissue on 3 sides with the final side, known as the pedicle, attached to provide an intact blood supply. The flap can then be advanced or rotated around the pedicle to cover the defect.

Unfortunately, flaps are subject to ischemia-induced complications that often result in partial or complete loss of the flap, necessitating further reconstructive efforts. This ischemia is believed to be the result of 2 factors: inadequate arterial perfusion and venous congestion due to poor venous output. Modalities aimed at improving flap survival have been the subject of intensive research. Improvement of patient risk factors, including smoking cessation, nutritional optimization, and control of diabetes, are well-established. Surgical techniques that favor flap survival, such as limited cautery and careful handling of the pedicle, have likewise been widely acknowledged. However, modalities that specifically target the flap have shown mixed results.

The first experiments specifically targeting flap perfusion focused on hyperbaric oxygen to increase oxygen delivery^1^^-^3 or leech therapy to relieve venous congestion.^4^^-^6 Pharmacologic agents, including vasodilators, anti-inflammatory drugs, and antioxidants,^7^ have been the focus of more recent studies. These agents have been injected at the flap site, injected intraperitoneally, applied as a mixture with fibrin glue, applied topically, or administered orally.^8^^-^12 At this time, despite extensive research into such drugs as nitric oxide,^13^ vascular endothelium growth factor and other growth factors,^14^ topical agents, and a variety of sympatholytics and vasodilators, the search for a drug that will reliably improve flap perfusion without significant side effects continues.

This study is designed to investigate a recently developed pharmacologic agent, tadalafil (Cialis: Eli Lilly, Indianapolis, Indiana), that has proven effective in increasing blood flow for patients with erectile dysfunction. Tadalafil, like similar drugs sildenafil (Viagra: Pfizer, NYC, New York) and vardenafil (Levitra: Bayer, Leverkusen, Germany), is a specific competitive inhibitor of cyclic guanosine monophosphate (cGMP) phosphodiesterase 5 (PDE-5) in vascular smooth muscle cells. Nitric oxide mediates vasodilatation through cGMP, and the inhibition of PDE-5 prolongs the dilatory effect. The drug has been shown to selectively increase blood flow to skeletal muscles and skin, while avoiding systemic hypotension. Through this mechanism, sildenafil has been shown to improve flap survival in rats^8^^-^10 and has made its way into clinical use. Of all the PDE-5 inhibitors, tadalafil has the longest half life at 17.5 hours thus decreasing necessary frequency of drug deliverance, with those of sildenafil and vardenafil being 4 hours and 5 hours, respectively.^15^ Local injection of tadalafil has been shown to improve flap survival in rats,^15^ but the ideal pharmacologic agent would be capable of oral administration for ease of postoperative care. Thus, this study aims to determine the potential benefit to skin flap healing and survival of an oral administration of tadalafil to experimental rats given standard skin flap surgeries. We hypothesized that oral administration of tadalafil should incur a comparable if not increased benefit in flap survival compared to sildenafil because of its similar mechanism and longer duration of action.

## MATERIALS AND METHODS

All experimentation was performed with the approval of the institutional review board and the Institution's Animal Care and Use Committee and was in compliance with all federal statutes regulating animal experimentation.

Thirty-seven male Sprague-Dawley rats (Harlan Laboratories, Indianapolis, IN) weighing 318 to 400 g were used in this experiment. All rats were allowed to acclimate for at least 1 week prior to surgery, housed in pairs in a standard animal facility with environmental control and a 12-hour light-dark schedule with regular food and water ad libitum. After surgery, the rats were housed individually to prevent injury to the surgical flap.

On the day of surgery, the animals were anesthetized with isoflurane induction via precision vaporizer and an attached scavenger system, 2% to 5% isoflurane by chamber initially, and then 1% to 3% by face mask for maintenance throughout the procedure. A modified McFarlane flap^16^ measuring 8 cm × 2.5 cm was raised with sharp dissection using a no. 10 scalpel blade using standard approved surgical procedure. The pedicle was placed caudally at the level of the iliac crests. The flap was then sutured back into its original donor position using 5-0 undyed monofilament sutures (Ethicon, Somerville, NJ) in a running subcuticular fashion. Analgesia was provided once preoperatively and postoperatively every 12 hours for 36 hours via a subcutaneous injection of 0.03 mg/kg buprenorphine.

Rats were divided into a control group, high-dose tadalafil group, and low-does tadalafil group. The control group was given the vehicle solution of 1% hydroxypropyl methylcellulose and 1% tween-80 in water. The test groups were given tadalafil powder (Eli Lilly and Company, Indianapolis, Indiana) mixed in the vehicle solution at either a high-dose concentration of 30 mg/mL for a dose of 20 mg/kg and the low-dose concentration of 15 mg/mL for a dose of 10 mg/kg. These concentrations were calculated on the basis of a 200 µL dose for a standard 300 g rat. Treatment doses were administered immediately before surgery and every 24 hours after surgery for a total of 7 doses. Study medication and control doses were administered by oral gavage using a bulb-tipped curved gavage syringe specifically designed for this purpose. The amount of treatment solution given was determined before each dosing based on the animal's weight at that time.

On postoperative days 7 and 14, the rats were anesthetized for tracing of the flap. A sheet of writable transparency paper was placed over the dorsum of each rat, accounting for the curvature of the dorsal surface, and the flap was traced taking care to draw the line of demarcation between the viable proximal flap tissue and the necrotic distal portion. Flap tracing analysis was performed digitally after each flap tracing was scanned into a computer as an image file. The images were then analyzed using Photoshop (Adobe, San Jose, California) to determine the percentage of necrosis and survival of each flap. A straight line was digitally drawn connecting the tracings representing the caudal ends of the lateral incisions. The areas of the resulting 2 enclosed shapes, representing the healthy and necrotic portions of the flap, were calculated by determining the number of pixels within each outline using the histogram function. Percentage of flap survival was determined by dividing the area of the healthy portion by the sum of the healthy and necrotic areas. Percentage of flap survival in each group was averaged and statistical analysis using the Mann-Whitney test was performed to determine the statistical significance of the data.

## RESULTS

All 37 surgeries were successful, and all rats continued to thrive until the end of the 14-day experimental period. By postoperative day 7, all flaps showed clear demarcation between the necrotic distal portion and the viable base.

**Figure 1:**
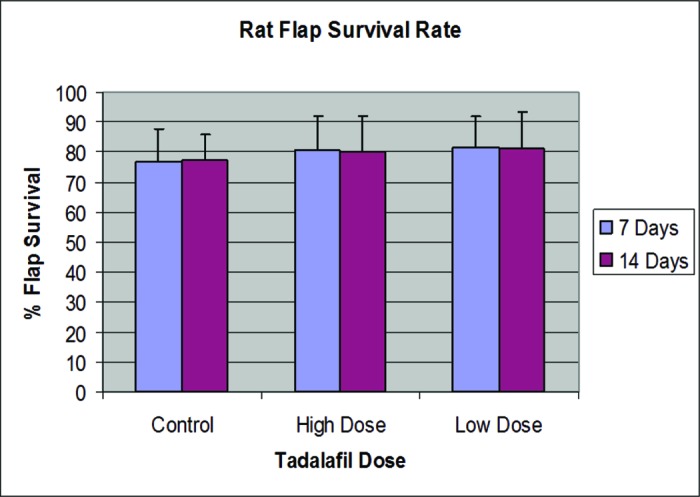
Flap survival rate.

The data for each of the experimental groups is shown in Table 1 and Figure 1. Control-group rats showed an average flap survival of 77% ± 11% at 7 days, and 77% ± 9% at 14 days. Low-dose-group rats showed an average flap survival of 82% ± 10% at 7 days (*P* = .21) and 81% ± 12% at 14 days (*P* = 0.41). High-dose-group rats showed an average flap survival of 81% ± 11% at 7 days (*P* = .45) and 80% ± 12% at 14 days (*P* = .53). Despite small trends in the data showing increased flap survival in the tadalafil treatment groups at days 7 and 14, these differences are not statistically significant based on analysis of variance with the Mann-Whitney test.

## DISCUSSION

Cutaneous flaps based on random blood supply are commonly used for the coverage of skin and soft tissue defects but are at high risk for ischemia and distal flap necrosis consequent to low axial blood flow. Pharmacologic agents that can increase blood flow and thereby improve flap survival could provide a low-risk method of minimizing and preventing such complications.

Phosphodiesterase-5 inhibitors are a family of agents that are most widely used to treat erectile dysfunction. Considering their ability to selectively increase blood flow to smooth muscle through the nitric oxide pathway, it has been hypothesized that they may be useful in improving perfusion to random pattern flaps. The first PDE-5 inhibitor to be tested on random pattern flap survival was sildenafil. It was shown to improve flap survival in a rat model through intraperitoneal administration,^8^^,^^9^ topically with a fibrin glue,^10^ and orally.^11^

Subsequently developed PDE-5 inhibitor, tadalafil, has a longer half-life and has been shown in studies of erectile dysfunction to be safe and effective with daily oral administration.^17^ Tadalafil was tested by Oh et al^15^ using a rat model with a local injection of the drug into the flap and demonstrated a significant improvement in random pattern flap survival. While injected administration of these drugs has shown promise, oral administration remains the most convenient method of drug deliverance. Our study showed a trend of increased flap survival in animals that received oral administration of tadalafil, but further studies are warranted to determine significance.

In our experience, we attribute the high standard deviation to several identified problems with the current study and studies like it. First, McFarlane flaps are very difficult to reproduce consistently. The dorsal rat skin is extremely mobile and very thick. The act of incising the skin frequently distorts the flap pattern. Second, administration of tiny doses of tadalafil is very imprecise. The amount of compound that is actually administered to the rats given the large fraction that remains in the dead space of the syringe and gavage needle is inconsistent. Third, transfer of the flap pattern and line of demarcated necrosis onto the transparency paper is approximate at best. Given these limitations, we suggest further study to examine the effect of daily oral administration of tadalafil. While our results are suggestive, they are not conclusive and warrant further investigation into the potential therapeutic use of the PDE-5 inhibitor, tadalafil.

## Figures and Tables

**Table 1:** Mean percent survival

	7 Days	*P*	14 Days	*P*
Control	76.86 (± 10.71)		77.16 (± 8.78)	
High dose	80.57 (± 11.43)	.45	80.32 (± 11.58)	.53
Low dose	81.79 (± 10.41)	.21	81.34 (± 12.28)	.41
